# Bridging the gap: neuroinflammation and the dawn of precision medicine in amyotrophic lateral sclerosis

**DOI:** 10.1186/s40035-026-00572-2

**Published:** 2026-08-24

**Authors:** Lu Tang, Dongsheng Fan

**Affiliations:** 1 Department of Neurology, Peking University Third Hospital, Beijing, China; 2 Beijing Key Laboratory of Innovative Diagnostics, Therapeutics and Translational Research for Neurological Rare Disorders, Peking University Third Hospital, Beijing, China

**Keywords:** Amyotrophic lateral sclerosis, Neurodegeneration, Neuroinflammation, Microglia, Immunotherapy, Biomarker, Neuroimmune endotype, Precision medicine

## Abstract

Neuroinflammation is no longer a secondary feature of amyotrophic lateral sclerosis (ALS), but rather a disease-modifying process that actively shapes the motor neuron vulnerability from the earliest stages of pathology. Central and peripheral immune cells, including microglia, astrocytes, and infiltrating T lymphocytes, adopt context-dependent states that can be neuroprotective or neurotoxic depending on disease stage and genetic background. These states are driven by discrete molecular programs, such as cGAS-STING-mediated innate immune sensing, NLRP3 inflammasome activation, and RIPK1-dependent necroptotic signaling, which represent tractable therapeutic targets. The repeated failure of broad-spectrum immunosuppressive trials reflects a fundamental mismatch between the non-selective interventions and the mechanistically distinct immune states of diseases. Converging transcriptomic, genetic, and immunophenotypic evidence supports the existence of putative neuroimmune endotypes in ALS, though this framework remains a working hypothesis pending prospective validation in biomarker-stratified cohorts. Advances in the following three domains are needed for realizing precision immunotherapy: standardized biomarker panels (including cerebrospinal fluid chitinases and TSPO-PET) to stratify patients by inflammatory subtype; pharmacodynamic readouts to confirm target engagement before interpreting clinical outcomes; and adaptive platform trial designs capable of evaluating mechanism-matching interventions in defined subgroups. This review integrates ALS-associated neuroinflammation with emerging precision medicine strategies, arguing that the central translational question is no longer whether or not to target neuroinflammation, but how, when, and in whom neuroinflammation should be targeted.

## Introduction

Amyotrophic lateral sclerosis (ALS) is a fatal neurodegenerative disorder characterized by progressive degeneration of both upper and lower motor neurons, leading to progressive muscle weakness, paralysis, and typically death from respiratory failure within 2 to 4 years after symptom onset [1]. Approximately 90% of cases are sporadic. The remaining 10% are familial, linked to mutations in genes such as *SOD1*, *C9orf72*, and *TARDBP* [2]. ALS is driven by multiple converging pathogenic mechanisms—including protein aggregation, mitochondrial dysfunction, oxidative stress, RNA dysregulation, and impaired nucleocytoplasmic transport—that vary in relative contribution across patients and disease stages, reflecting profound genetic and molecular complexity of ALS [1]. Increasingly, it has become evident that disease evolution is not solely neuron-autonomous, but critically shaped by interactions between motor neurons and the immune system [3].

The role of neuroinflammation in ALS has evolved from being a mere “bystander” to a recognized disease driver. Compelling evidence indicates that inflammation, involving central innate immune activation and infiltration of peripheral immune cells into the central nervous system (CNS), plays a role in ALS pathophysiology [4–6]. Microglial activation is detectable not only in symptomatic patients but also in presymptomatic *SOD1* mutation carriers, suggesting that neuroimmune alterations precede overt neurodegeneration [7]. Postmortem analyses further demonstrate widespread astrogliosis and microgliosis in the motor cortex and spinal cord [8], accompanied by infiltration of peripheral immune cells, including macrophages, T lymphocytes, and natural killer (NK) cells [9]. Importantly, alterations in adaptive immunity, most notably a reduction in regulatory T cells (Tregs), correlate with faster disease progression [10], underscoring the functional relevance of immune imbalance in ALS. Given its central role, a precise understanding of “neuroinflammation” itself is warranted.

It is critical to distinguish neuroinflammation within the broader field of neuroimmunology. Neuroimmunology encompasses all bidirectional nervous–immune interactions, including physiological immune surveillance. Neuroinflammation [11] specifically refers to chronic, pathological activation of CNS-resident glia (microglia and astrocytes), accompanied by inflammatory factor release, tissue damage, and often infiltration of peripheral immune cells. Neuroinflammation functions as a “double-edged sword”, essential for homeostasis and repair initially, while also capable of driving chronic toxicity when dysregulated [12]. In ALS, this process is a key driving mechanism and a critical nexus connecting neuroimmune dysfunction to motor neuron degeneration.

Despite this compelling body of evidence, translation of neuroinflammatory insights into effective therapies has proven remarkably difficult. Clinical trials employing broad-spectrum immunosuppressive or anti-inflammatory agents have repeatedly failed to modify disease course [13], revealing a critical disconnect between biological understanding and therapeutic strategy. These failures do not negate the importance of neuroinflammation; rather, they reflect a fundamental design flaw, i.e., applying non-selective immune suppression to a disease in which the inflammatory milieu shifts across disease stages and differs between genetic subgroups, and protective versus toxic effectors balance also varies. Neuroinflammation in ALS comprises distinct, context-dependent immune states that may exert protective or toxic effects depending on disease stage, genetic background, and cellular milieu.

Collectively, these features are consistent with the putative neuroimmune endotypes in ALS, which are biologically distinct immune states that may differentially respond to mechanism-specific interventions. This concept provides a compelling conceptual framework for precision therapeutic stratification and for determining how, when, and in whom the immune pathways should be modulated. 

## Neuroinflammation in ALS

**Fig. 1:**
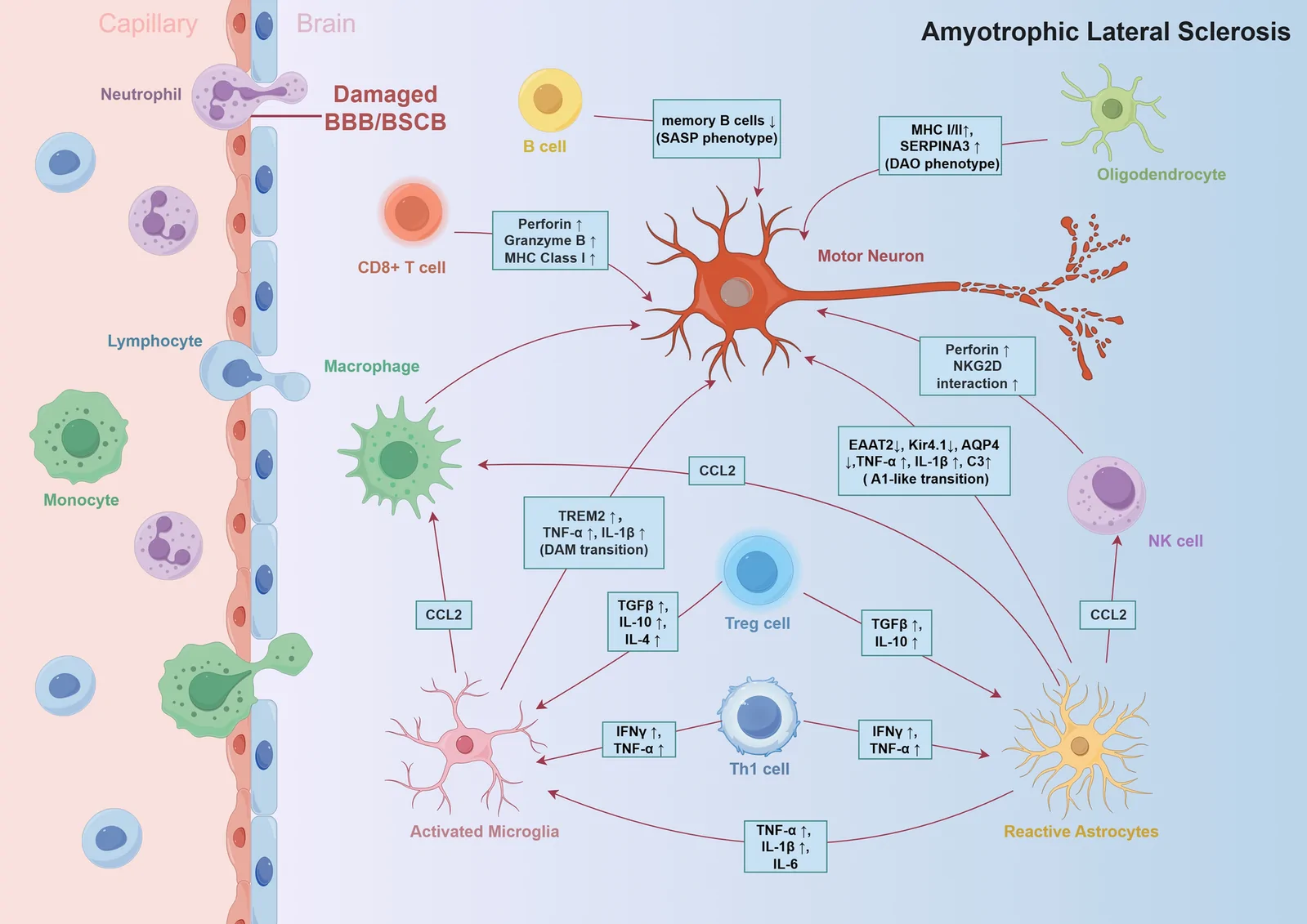
Schematic illustrating the complex interplay between the central nervous system (CNS) residents and infiltrating peripheral immune cells in ALS. The breakdown of blood–brain barrier (BBB) and blood-spinal cord barrier (BSCB) facilitates the infiltration of peripheral immune cells into the CNS. Infiltrating neutrophils, monocytes, and T cells interact with resident glial cells to establish a self-sustaining inflammatory cycle. Monocytes differentiate into macrophages, while CD8^+^ T cells release perforin and granzyme B, directly targeting motor neurons expressing major histocompatibility complex (MHC) class I. B cells exhibit a senescence-associated secretory phenotype (SASP). Inside the CNS, this cross-talk drives microglia toward a disease-associated microglia (DAM) phenotype (TREM2^+^, IL-1β^+^) and astrocytes toward a neurotoxic A1-like state (C3^+^, TNF-α^+^), both of which contribute to motor neuron degeneration. Oligodendrocytes display a disease-associated oligodendrocyte (DAO) phenotype characterized by upregulation of SERPINA3 and MHC class I/II. Natural killer (NK) cells exert cytotoxicity via NKG2D interactions. Conversely, the protective capacity of regulatory T (Treg) cells is compromised, failing to suppress the pro-inflammatory milieu mediated by interferon-gamma (IFN-γ) and tumor necrosis factor-alpha (TNF-α)

In ALS, immune responses can be divided in two main subtypes: glial neuroinflammation [14, 15] and activation of the peripheral immune system [16]. The immune system exists in a balance between protective and inflammatory factors, with an imbalance linked to pathogenesis and progression of ALS [3]. Together, these cell-type-specific and context-dependent inflammatory programs provide a biological rationale for hypothesizing distinct neuroimmune endotypes in ALS, i.e., immune states defined by the predominance of particular cellular and molecular programs, rather than a uniform pathogenic mechanism (Fig. 1). Whether such endotypes can be prospectively identified and clinically implemented remains an active area of investigation.

### Central innate immune cells in ALS

The immune response within the CNS involves both local innate components and transported elements from the periphery [17]. The innate immune response is primarily coordinated by resident glial cells, notably microglia and astrocytes. Once considered merely supportive, these cells are now recognized as active and dynamic regulators of neuronal health, inflammation, and disease progression in ALS. Their roles are complex, often dichotomous, and evolve across different disease stages.

#### Microglia: the double-edged sword of neuroinflammation

Microglia, which originate from primitive macrophages in the mesoderm-derived yolk sac, colonize the CNS during developmental stages [18]. As the resident immune cells of the CNS and the first responders to pathological insults, microglia serve as the first line of defense for the brain and spinal cord. They monitor the microenvironment to detect signs of damage, protect neurons from infection or injury, and regulate synaptic pruning [19].

In ALS, misfolded proteins (such as mutant SOD1[mSOD1] [20] and TAR DNA-binding protein 43 [TDP-43] [21]) are key activators of microglia, engaging receptors including CD14, Toll-like receptors (TLRs) 2 and 4, scavenger receptors [22], and the NOD-like receptor family pyrin domain containing 3 (NLRP3) inflammasome [23]. Upon activation, microglia undergo complex and dynamic phenotypic and functional shifts that are critical for motor neuron fate [24, 25]. Notably, spatial transcriptomic analysis of microglia in SOD1 models revealed that changes in microglial gene expression precede motor neuron pathology and symptom onset [26, 27], implicating microglia in non-cell autonomous neurotoxicity. This concept is supported by evidence that modulating mSOD1 expression specifically within motor neurons is insufficient to influence disease onset or progression [28, 29]. By contrast, selective silencing of mSOD1 in microglia significantly delays motor neuron degeneration and extends survival [29]. Consistently, extended survival has been observed after transplantation of wild-type non-neuronal cells into mSOD1 chimeric mice [30]. Therefore, deciphering this complex interplay between microglial activation and motor neurons in ALS is crucial for developing therapeutics aiming at modulating microglial function to halt or slow disease progression.

During ALS, microglia undergo a dynamic phenotypic shift, a process better described as a continuous activation spectrum than the oversimplified M1/M2 dichotomy. In presymptomatic and early disease stages of ALS, microglia typically exhibit a neuroprotective (previously described as M2-like) phenotype [31], characterized by increased expression of homeostatic markers (such as CD206 and TREM2) that contribute to tissue repair and neuronal survival. Functionally, they phagocytose protein aggregates (e.g., misfolded SOD1, TDP-43) [32, 33], clear cellular debris [34], and release anti-inflammatory cytokines (e.g., interleukin [IL]-4, IL-10, TGF-β) [35] and neurotrophic factors (e.g., IGF-1, brain-derived neurotrophic factor) [36]. With sustained pathological stimuli, they transition to a disease-associated microglial (DAM) state [37]. Morphologically, the cells become amoeboid and cluster around degenerating motor neurons, with upregulated expression of apolipoprotein E and IL-1β [38, 39]. Critically, this shift involves a dual detriment: a gain of neurotoxic function through the release of pro-inflammatory mediators (e.g., TNF-α, IL-1β, IL-6) and reactive species (e.g., Reactive oxygen species [ROS], NO), and a loss of homeostatic function, including impaired phagocytosis.

The role of microglia in ALS pathogenesis is further highlighted by genetics. *C9orf72*, the most commonly mutated gene in familial ALS and frontotemporal dementia, is highly expressed in myeloid cells including microglia [40]. Loss-of-function mutations in *C9orf72* lead to haploinsufficiency, which disrupts lysosomal function, impairs autophagy, and can lead to hyperactivation of the cyclic GMP-AMP synthase (cGAS)-stimulator of interferon genes (STING) innate immune pathway in microglia [41]. This impairment of cellular “housekeeping” functions not only reduces the clearance efficiency, but also promotes a hyperinflammatory state. Consistently, *C9orf72* inactivation in mice triggers microglial abnormalities and age-related neuroinflammation [42] (Fig. 1).

#### Astrocytes: from supportive partners to toxic executioners

Astrocytes orchestrate CNS homeostasis through metabolic support, ion buffering, neurotransmitter recycling, and blood–brain barrier maintenance. In ALS, they become reactive (astrogliosis)[43]. Studies have shown that astrocytes expressing ALS-linked mSOD1 are toxic to motor neurons both in vitro and in vivo [44, 45]. Selectively silencing the mSOD1 gene in astrocytes or transplanting healthy astrocytes reduces the toxicity, decreases motor neuron loss, delays disease progression, and extends the lifespan of mSOD1 mice [44, 45]. Conversely, transplanting astrocytes expressing mSOD1 induces focal motor neuron degeneration and death in the spinal cords of wild-type rats [46]. Furthermore, astrocytes reprogrammed from fibroblasts of ALS patients impair the survival of motor neurons [47]. Therefore, the expression of ALS-associated mutant proteins in astrocytes confers non-cell autonomous toxicity.

Astrocytes contribute to pathogenesis through both loss of normal supportive functions and gain of toxic functions [48, 49]. Pronounced downregulation of the astrocytic glutamate transporter excitatory amino acid transporter 2 (EAAT2, or GLT-1 in rodents) leads to impaired synaptic glutamate clearance and results in chronic neuronal excitotoxicity, calcium overload, and motor neuron death [50, 51]. Concurrently, neuronal glycogen stores are limited, necessitating dependency on the astrocyte-neuron lactate shuttle for continuous energy supply. The core of this system is the spatiotemporal cooperation between the astrocyte lactate transporter MCT4 and the neuronal transporter MCT2 [52, 53]. In ALS, astrocytes with decreased MCT1 levels may fail to provide sufficient lactate or other energy substrates to metabolically stressed motor neurons [54]. Moreover, the loss of the inward-rectifying potassium channel Kir4.1 from astrocytes impairs extracellular K⁺ buffering, contributing to neuronal hyperexcitability [49, 55, 56]. This is compounded by a disruption in the polarized localization of aquaporin-4 at astrocytic endfeet, which perturbs water homeostasis and glymphatic waste clearance, fostering a toxic microenvironment [57].

Beyond these deficits, reactive astrocytes actively acquire neurotoxic functions [58]. They release pro-inflammatory cytokines (e.g., TNF-α, IL-1β), ROS, and specific toxic molecules that directly harm motor neurons [58, 59]. Recent studies have revealed that astrocytes can trigger motor neuron death by activating necroptosis, a regulated, caspase-independent form of cell death mediated by the receptor-interacting serine/threonine-protein kinase 1 (RIPK1)/mixed lineage kinase domain-like (MLKL) pathway [14]. This specific, druggable cell-death mechanism initiated by astrocytes represents a promising new therapeutic avenue (Fig. 1).

#### Oligodendrocytes (OLs)

OLs, once considered solely as passive myelinating and supportive cells [60], are now recognized as dynamic contributors to neuroinflammation in ALS [61]. Central to this shift is the acquisition of a disease-associated oligodendrocyte (DAO) phenotype [62]. OL lineage cells aberrantly upregulate major histocompatibility complex class I/II (MHC-I/II) molecules [63], having antigen-presenting capacity to potentially activate infiltrating CD4^+^ and CD8^+^ T cells. This is complemented by the expression of pro-inflammatory genes such as *SERPINA3* [64]. Critically, this immune-like transformation is driven in part by TDP-43 proteinopathy [65], which disrupts crucial cellular functions of OLs.

Furthermore, OLs engage in complex bidirectional inflammatory crosstalk with microglia and astrocytes [61], forming a vicious inflammatory cycle. Activated astrocytes and microglia release mediators like CHI3L1 [66] and TNF-α [67], which potently inhibit oligodendrocyte precursor cell (OPC) differentiation and remyelination. Conversely, the diseased OLs and DAOs release signals that promote a pro-inflammatory, activated phenotype of nearby microglia. A previous study [68] confirmed that the loss of OPCs leads to a concomitant loss of the homeostatic gene signature in microglia, highlighting their tight functional coupling.

Finally, OL dysfunction and neuroinflammation can exacerbate each other. For instance, lipid metabolism dysregulation in OLs (e.g., impaired cholesterol synthesis) caused by TDP-43 proteinopathy [65] not only directly leads to myelin damage and demyelination, but the myelin debris itself acts as a potent inflammatory stimulus, continuously activating microglia and astrocytes [60]. Simultaneously, the collapse of OL metabolic support (e.g., lactate transport) results in axonal energy stress and injury, further releasing damage-associated molecular patterns (DAMPs) that aggravate the local inflammatory response [61] (Fig. 1).

### Infiltration of the peripheral immune system

In ALS, the peripheral immune system is not merely reactive, but is a major driver of disease progression [69, 70]. This is characterized by alterations in immune cell populations, dysfunctional immune cell activity, and a systemic shift toward a pro-inflammatory cytokine profile—a phenomenon observed in both patients and animal models [16].

The pathology of ALS extends far beyond motor neurons [71]. A central feature is the loss of functional integrity in the blood–brain barrier (BBB) and blood-spinal cord barrier (BSCB). While healthy CNS is immune-privileged, with minimal infiltration of peripheral immune cells [72], breakdown of these barriers [73] in ALS facilitates the infiltration of T cells [74, 75], monocytes/macrophages [5, 76], and NK cells [77], likely due to a combination of early glial activation, inflammatory factor accumulation, or vascular abnormalities.

Concurrently, degenerating motor neurons and activated glial cells release DAMPs [78], abnormal protein aggregates [65](e.g., TDP-43), and various chemokines [79, 80](e.g., C–C motif chemokine ligand 2 [CCL2]/monocyte chemoattractant protein-1 [MCP-1], C-X3-C motif chemokine ligand 1 [CX3CL1]). These signals establish a strong chemotactic gradient, actively recruiting peripheral immune cells to the disease frontier within CNS. This process tightly couples local CNS pathology with the systemic immune response, making neuroimmune dysfunction a core component of ALS pathogenesis (Fig. 1).

#### T lymphocytes: orchestrating immune imbalance

The infiltration of T lymphocytes into the spinal cord and motor neuron injury sites is a well-documented feature of ALS, signifying a pivotal role for the adaptive immune system in disease pathogenesis. The functional balance between distinct T cell subsets critically shapes the neuroinflammatory microenvironment and influences clinical decline. In experimental models such as SOD1^G93A^ mice, the infiltration follows a temporal pattern, with early appearance of CD4^+^ T cells and later presence of both CD4^+^ and CD8^+^ T cells [81].

CD4^+^ helper T cells [82], comprising pro-inflammatory Th1 and Th17 subsets [83] as well as anti-inflammatory Th2 cells and Tregs, are central regulators of immune dysregulation [74]. The pathogenic shift towards a pro-inflammatory state is largely driven by cytokines such as interferon-gamma (IFN-γ, from Th1 cells) and IL-17 (from Th17 cells), which potently activate microglia and astrocytes, thereby exacerbating oxidative stress and neurotoxicity. Single-cell transcriptomic atlases of cerebrospinal fluid (CSF) and peripheral blood indicated that the distribution of T cell subsets is broadly consistent between peripheral blood and CSF in ALS patients [84], reflecting the characteristics of the CSF immune microenvironment. A key aspect of this imbalance is the loss of protective regulation. Tregs, defined by expression of Foxp3 and CD25 [75, 85, 86], suppress excessive inflammation via IL-10 and TGF-β secretion. In ALS, both the frequency and the suppressive function of Tregs are significantly diminished in the peripheral blood and CSF [75, 87], a deficit strongly correlated with rapid progression. This finding underscores a compelling model where disease acceleration results from both an increased pro-inflammatory drive and a failure of endogenous immunosuppression.

CD8^+^ cytotoxic T cells are found in increased densities within the motor cortex and spinal cord parenchyma in ALS model mice [81, 88], often proximal to neurons and astrocytes. Clonally expanded CD8^+^ effector memory T cells identified in patient CSF share identical receptors with blood counterparts, implying active CNS trafficking and a targeted immune response [84]. While direct evidence of neuron killing in vivo remains limited, these cells possess cytotoxic machinery (e.g., perforin, granzymes) [88, 89], potentially targeting stressed neurons or facilitating the removal of damaged cells (Fig. 1).

#### B lymphocytes: a pivotal node of functional polarization

B lymphocytes play a complex and increasingly recognized dual role in ALS immunopathology, acting as active participants rather than bystanders. In patients, B cells exhibit significant alterations linked to immunosenescence. Elevated frequencies of “late/exhausted memory B cells” in peripheral blood correlate with adverse clinical features such as rapid progression and bulbar onset [90]. These cells display a senescent phenotype [91] and may exacerbate local and systemic inflammation via senescence-associated secretory phenotype [92]. Critically, multivariate analyses identify high baseline levels of these cells as an independent risk factor for shorter survival, suggesting that they are active drivers of pathology rather than mere biomarkers.

Conversely, preclinical studies demonstrate a protective potential of certain B cell subpopulations. In SOD1^G93A^ mice [93], infusion of healthy naïve or regulatory B cells (Bregs) [94] delayed disease onset, improved motor function, and extended survival. The treatment reduced spinal neuron apoptosis and astrogliosis while shifting the systemic immune profile toward an anti-inflammatory state. This beneficial remodeling, termed “pligodraxis” [93], highlights the capacity of infused B cells to reshape the immune milieu. A preliminary human case report found that B cell infusion in an advanced ALS patient [93] safely modulated systemic immunity, reducing pro-inflammatory cytokines (e.g., IL-6, TNF-α) and altering leukocyte subsets with transient functional improvement. This foundational work supports the ongoing exploration of Breg-targeted therapies in early-phase clinical trials. Future therapeutic strategies may therefore need a dual approach: targeting pathogenic B cell clones while harnessing the regulatory capacity of Bregs [94, 95] for precision immunomodulation (Fig. 1).

#### Monocytes/Macrophages: compromising barriers and fueling inflammation

Monocytes serve as a crucial link between peripheral and central inflammation in ALS [5, 96]. Upon CNS infiltration and differentiation into macrophages, they potently amplify inflammatory response [97]. Beyond exacerbating neuroinflammation, specific subsets, such as perivascular macrophages (PVMs), directly compromise the integrity of the CNS barriers [98, 99].

Monocyte heterogeneity has clinical implications. For instance, one study found that a higher baseline frequency of non-classical monocytes expressing CD11b predicts shorter survival in ALS patients [100]. Furthermore, monocytes from rapidly progressing patients and ALS mice display a transcriptional profile skewed toward a pro-inflammatory state with enhanced migratory capacity, facilitating their infiltration into the CNS [5, 96].

Recruited by chemokines like CCL2, monocytes differentiate into macrophages [98]. PVMs, located at the blood vessel–parenchyma interface, are key cellular components of the BSCB. These newly differentiated macrophages closely resemble activated microglia in both morphology and function. In SOD1^G93A^ mice [5], the number of PVMs increases with disease progression, and the proportion of the pro-inflammatory MHCII^+^ subtype rises. Notably, selective and sustained depletion of PVMs can prevent motor neuron loss, slow disease progression, and extend survival by reducing extracellular matrix degradation and preserving the BSCB integrity [101]. These findings establish PVMs as a promising therapeutic target [98, 99] in ALS (Fig. 1).

#### Neutrophils: prognostic sentinels and putative effectors

Neutrophils are rapid innate immunity responders. Emerging evidence suggests that they are not merely systemic inflammatory markers of ALS, but actively contributors to the disease pathogenesis [102, 103].

Peripheral blood profiling of ALS patients often revealed a relative increase in neutrophils alongside a decrease in lymphocytes, resulting in a significantly elevated neutrophil-to-lymphocyte ratio [104], reflecting an imbalance between innate and adaptive immunity. Studies have confirmed that a high baseline neutrophil-to-lymphocyte ratio can independently predict shorter survival in ALS patients [105]. For example, a study stratified patients by neutrophil-to-lymphocyte ratio and demonstrated that patient with a high neutrophil-to-lymphocyte ratio experienced faster progression and reduced survival [106]. Additionally, an elevated baseline neutrophil count and increased surface expression of the activation marker CD16 (FcγRIII) correlate with more rapid disease progression [69]. Collectively, these findings highlight that a constitutive pro-inflammatory immune status drives aggressive ALS.

Neutrophils may exacerbate ALS pathology through several interrelated mechanisms. First, upon CNS infiltration, they can directly inflict tissue damage by releasing cytotoxic mediators such as ROS, myeloperoxidase [107, 108], and neutrophil extracellular traps [109], amplifying local neuroinflammation. Second, neutrophil-secreted matrix metalloproteinases [110, 111] degrade extracellular matrix and basement membrane components, compromising the BBB/BSCB integrity and facilitating further immune cell infiltration (Fig. 1).

#### Natural killer (NK) cells: activated cytotoxic participants

NK cells contribute to ALS progression through direct cytotoxicity and immunoregulation [100]. Flow cytometry studies have confirmed that increased peripheral NK cell levels correlate with disease severity [69, 112]. NK cells are commonly divided into immunoregulatory CD56^bright^ and cytotoxic CD56^dim^ subsets [113]. Recent single-cell RNA sequencing analyses further revealed that in sporadic ALS patients, the terminally differentiated, highly cytotoxic CD56^dim^ subset (NK_2) is substantially expanded [77], exhibiting altered gene expression profiles and cell–cell communication patterns linked to immune activation.

Under pathological conditions, NK cells contribute via multiple mechanisms [114]. Their infiltration into the CNS has been detected in postmortem motor cortex and spinal cord tissues of ALS patients [115], as well as in CNS tissues of SOD1^G93A^ and TDP-43^A315T^ models, with early recruitment of CCL2 signaling [100]. In pathological conditions, damaged motor neurons express surface NKG2D ligands [100], rendering them susceptible to NK cell recognition[11]. NK cells can directly induce motor neuron death via perforin and granzymes release. Beyond direct cytotoxicity, they also modulate other immune cells. Studies in SOD1^G93A^ mice showed that NK cells interacte with microglia and Tregs [100]. NK cell depletion delayed motor neuron loss, altered microglial activation, promoted Treg infiltration, and extended survival [116]. Targeting NK cells or their functional pathways, such as the JAK/STAT signaling pathway [117], is an active research direction, though the precise mechanisms and clinical potential require further elucidation [118].

In summary, central and peripheral immune systems converge into a self-sustaining inflammatory cycle in ALS. Early barrier disruption allows peripheral monocytes, T cells, and neutrophils to infiltrate the CNS, guided by chemokines and DAMPs released from activated glia and stressed neurons. Inside the CNS, infiltrating Th1/Th17 cells and macrophages interact with microglia and astrocytes, promoting a cytotoxic state via mediators like TNF-α and IL-1β, while protective Treg activity declines. This inflammatory response further damages barriers and neurons, facilitating immune recruitment. Thus, a localized injury escalates into a chronic, systemic neuroinflammatory process, which is a key target for therapeutic intervention (Fig. 1).

### Core inflammatory signaling pathways

The core inflammatory signaling pathways in ALS constitute a complex regulatory network [16]. They serve not only as the molecular foundation for the activation and function of various immune cells, including microglia, astrocytes, and peripherally infiltrating cells, but also as a critical bridge linking upstream pathogenic factors (such as mutant proteins) to downstream neuronal death.

#### Pattern recognition receptor (PRR) pathways: from causal drivers to nonspecific amplifiers

PRRs constitute the core surveillance and signaling system of the innate immune system [119]. In ALS, PRR-associated transcriptional and cytokine programs are frequently observed across patient tissues and experimental models, consistent with a role in the ignition and amplification of neuroinflammation [120]. Expressed on CNS innate immune cells (e.g., microglia, astrocytes), PRRs recognize both pathogen-associated molecular patterns and host-derived DAMPs [121]. This recognition triggers intracellular signal transduction cascades, leading to the production of pro-inflammatory cytokines, chemokines, and type I interferons. Rather than functioning as a monolithic inflammatory switch, PRR signaling in ALS operates across a hierarchy of specificity and causality. Distinguishing primary, genotype-driven triggers from secondary, nonspecific amplifying responses is essential for prioritizing therapeutic targets.

##### cGAS-STING pathway: a genotype-defined inflammatory driver

In contrast to the nonspecific nature of traditional inflammatory pathways, the cytosolic DNA sensing via the cGAS-STING pathway represents a compelling, genetically relevant inflammatory driver, with particularly mechanistic relevance in genetically defined ALS subgroups—most notably *C9orf72* loss-of-function and TDP-43 pathology cases—where upstream triggers of pathway activation are directly linked to disease-causing mutations. Activation of this pathway is tightly linked to specific ALS genotypes through defined molecular mechanisms. *C9orf72* haploinsufficiency impairs autophagy/lysosomal function, leading to cytosolic DNA accumulation and STING hyperactivation [122]. Cytoplasmic mitochondrial DNA released due to TDP-43 pathology or general mitochondrial dysfunction serves as a potent DAMP for cGAS, resulting in TANK-binding kinase 1 (TBK1) and IRF3 (interferon regulatory factor 3) activation and subsequent type I interferon induction [78], as well as nuclear factor kappa-B (NF-κB)-mediated expression of cytokines like IL-6 [123]. Additionally, epigenetic derepression (reactivation) of human endogenous retroviruses (e.g., HERV-K) in ALS [124] leads to reverse-transcribed cytoplasmic double-stranded DNA that can activate cGAS. Emerging evidence further suggests cell-autonomous activation of this pathway within vulnerable motor neurons themselves [125]. Experimentally, blockade of STING mitigates inflammation and neurodegeneration in both iPSC-derived neuronal cell models and ALS/FTD- and aging-relevant mouse models [78, 123, 126], highlighting its therapeutic potential.

##### Toll-like receptor 4 (TLR4) pathway: the broad-spectrum upstream sensor

While cGAS-STING responds to specific intracellular genetic and mitochondrial stress, TLR4 acts as a broad-spectrum sensor of extracellular alarmins. Extracellular aggregates of disease-related proteins like misfolded SOD1 and TDP-43 [127] can act as DAMPs and can engage TLR4 on microglia and astrocytes, signaling typically via the adaptor MyD88 to activate NF-κB [128, 129]. This drives the expression of key pro-inflammatory mediators such as TNF-α, IL-1β, and IL-6, fueling a sustained inflammatory cascade [130]. In ALS models, TLR4 deficiency ameliorates disease progression but does not prevent onset, indicating it acts as a secondary amplifier rather than a primary disease-initiating event [127]. Combined with the functional redundancy of innate immune sensing, this likely limits the therapeutic efficacy of targeting TLR4 alone.

##### NLRP3 inflammasome pathway: the convergent terminal amplifier

Downstream of both intracellular DNA sensing (cGAS-STING) and extracellular alarmin detection (TLR4), the NLRP3 inflammasome functions as a terminal integration node and inflammatory amplifier. NLRP3 inflammasome activation follows a canonical “two-signal process” [131]: a priming signal (Signal 1) — provided by NF-κB activation downstream of PRRs such as TLR4 and indirectly by IFN signaling downstream of the cGAS-STING axis — to induce the expression of NLRP3 and pro-IL-1β; and an activation signal (Signal 2) triggered by disease-associated cellular stressors, most commonly K⁺ efflux, as well as mitochondrial ROS and lysosomal disruption caused by protein aggregates [131]. In the ALS context, pathological ALS proteins (including mSOD1 and TDP-43) activate the microglial NLRP3 inflammasome via ROS and ATP release, leading to caspase-1 activation, ASC (apoptosis-associated speck-like protein containing a CARD) formation, and IL-1β secretion [23]. Consistent with this, NLRP3 together with ASC, active caspase-1, IL-1β and IL-18 are upregulated in SOD1 mice and in post-mortem tissue from sporadic ALS patients, with components already detectable at pre-symptomatic stages [120, 132]. Notably, caspase-1 activation also triggers the gasdermin D-mediated pyroptosis, a lytic form of inflammatory cell death that further releases inflammatory contents into the extracellular space [133]. Because these upstream stressors are common pathological features across diverse neurodegenerative conditions, NLRP3 inflammasome serves as a convergent amplifier that integrates heterogeneous ALS-associated signals into a unified IL‑1β/IL‑18‑ and pyroptosis‑driven inflammatory output. However, NLRP3 is best viewed as a downstream effector rather than a primary disease driver, as NLRP3 inhibition alone is insufficient to suppress spinal cord inflammation in ALS models when multiple inflammasomes such as NLRP1 and NLRC4 are engaged [134].

#### Cytokine signaling pathways: the “amplifier” and “executioner” of inflammation

Persistent elevation of cytokines in the CSF of ALS patients has been observed [135]. However, broad cytokine-targeting therapies have all failed [13]. This suggests that the cytokine networks in ALS are not uniformly toxic, but are temporally dynamic and intersect directly with core disease-specific pathomechanisms.

Mature IL-1β, produced largely via the NLRP3 and other inflammasomes, binds to IL-1 receptors on neurons and glia [5, 136]. Beyond amplifying inflammation, IL-1β directly compromises neuronal survival by reducing astrocytic glutamate uptake, at least in part by promoting PKC- and dynamin-dependent endocytosis of GLT-1 and GLAST (glutamate-aspartate transporter) [50]. Additionally, in the ALS environment, EAAT2 (GLT-1 in rodents) is cleaved by caspase-3 at its C-terminus [137, 138], further impairing glutamate clearance and exacerbating excitotoxic damage.

Similarly, the role of TNF-α depends on disease stage rather than its absolute concentration. While acute TNF-α signaling via TNF receptor 2 (TNFR2) supports neuroprotection and myelin maintenance, deleterious and chronic TNF-α increase in the ALS spinal cord shifts the signaling toward TNF receptor 1 (TNFR1), thereby initiating the RIPK1/MLKL-mediated necroptosis [14, 139, 140]. This temporal shift — from protective to lethal — provides a mechanistic rationale against the broad anti-TNF-α strategies, which could indiscriminately abolish residual TNFR2-mediated homeostatic signaling alongside the pathogenic TNFR1 drive.

Importantly, the clinical relevance of these cytokines is underscored by consistent findings in human patients: both IL-1β and TNF-α are significantly elevated in the CSF of ALS patients compared to controls, as demonstrated by a systematic review and meta-analysis encompassing 2629 patients across 71 studies [135], and CSF IL-1β levels are negatively correlated with ALS functional rating scale-revised (ALSFRS-R) scores [141].

In contrast to these CNS-resident effectors, IFN-γ is secreted by infiltrating Th1 and NK cells [74, 89]. IFN-γ forces microglial polarization toward a neurotoxic, ROS- and nitric oxide-releasing phenotype via JAK-STAT1 activation [100, 142, 143]. Rather than merely correlating with BBB disruption, IFN-γ actively translates peripheral immune infiltration into amplified central toxicity, providing a mechanistic rationale for ongoing JAK/STAT inhibition trials aimed at decoupling the periphery from the CNS.

#### Chemokine pathways: the “navigation system” for immune cells

Chemokine pathways guide immune cell trafficking like a “navigation system”. The CCL2 (MCP-1)–CCR2 (C–C chemokine receptor type 2) axis is a central regulator of monocyte infiltration. Activated microglia and astrocytes in the ALS produce CCL2 [9, 80], which binds to CCR2 on circulating monocytes [144]. This signaling directs monocyte chemotaxis into the CNS and neuromuscular junction (NMJ) regions, compromises BBB/BSCB integrity, and drives neuromuscular denervation in both ALS patients and mouse models [145]. Meanwhile, under homeostatic conditions, neuron-derived CX3CL1 (fractalkine) engages CX3CR1 on microglia to maintain a surveillance state [146]. In ALS, this communication is disrupted, with altered expression and signalling of CX3CL1/CX3CR1 [147, 148], contributing to aberrant microglial activation and neuronal damage.

#### The complement system: from synaptic pruning to inflammation

The complement system, as an intricate part of the innate immune system, is aberrantly activated in ALS [149, 150]. Early in disease, the initiator component C1q [151], which is primarily produced by microglia, deposits onto vulnerable synapses and neuromuscular junctions, thereby tagging them for microglial phagocytosis — a process analogous to pathological synaptic pruning. C5a is a terminal component of complement activation [152] and a potent anaphylatoxin. By binding to its receptor C5aR1, C5a robustly drives the recruitment and activation of immune cells and synergistically amplifies inflammation, including that mediated by TLR signaling. Therefore, complement activation is directly linked to the escalation of neuroinflammation in ALS.

#### The receptor-interacting protein kinase 1 (RIPK1) pathway: a promising therapeutic target

RIPK1 is a serine/threonine protein kinase that has emerged as a compelling therapeutic target for ALS [140]. It integrates upstream signals from receptors like TNFR1. RIPK1 function is intricately regulated by cellular energy status, post-translational modifications, and the microenvironment, contributing to cell-fate decisions toward survival, apoptosis, or necroptosis. In ALS, multiple genetic and environmental factors converge to drive aberrant RIPK1 activation, which acts as a key node to simultaneously amplify two core pathogenic pathways: neuroinflammation and neuronal death (in part via necroptosis) [139, 153].

RIPK1 possesses dual functions: its scaffolding role supports the pro-survival NF-κB signaling, whereas its kinase activity can initiate RIPK1-dependent apoptosis or necroptosis [139, 154]. Notably, in ALS models, activated RIPK1 promotes the gliosis-mediated release of TNF-α and IL-1β. Its activity is tightly regulated by ubiquitination and phosphorylation. Mutations in ALS-linked genes such as *TBK1* [155] and *OPTN* [156] impair this regulation, leading to excessive RIPK1 kinase activity in experimental models. Single-cell sequencing has identified a distinct microglial subset termed RIPK1-regulated inflammatory microglia (RRIMs) [157]. In ALS models, RIPK1 inhibition reduces the RRIM prevalence, showing therapeutic potential. These findings support the hypothesis that RRIMs represent a mechanistic link between RIPK1 activity and neuroinflammation, although a direct causal relationship between RRIM reduction and functional benefit remains to be established. Structurally, the presence of an allosteric pocket in its kinase domain allows for highly selective inhibition by small molecules [158].

**Fig. 2:**
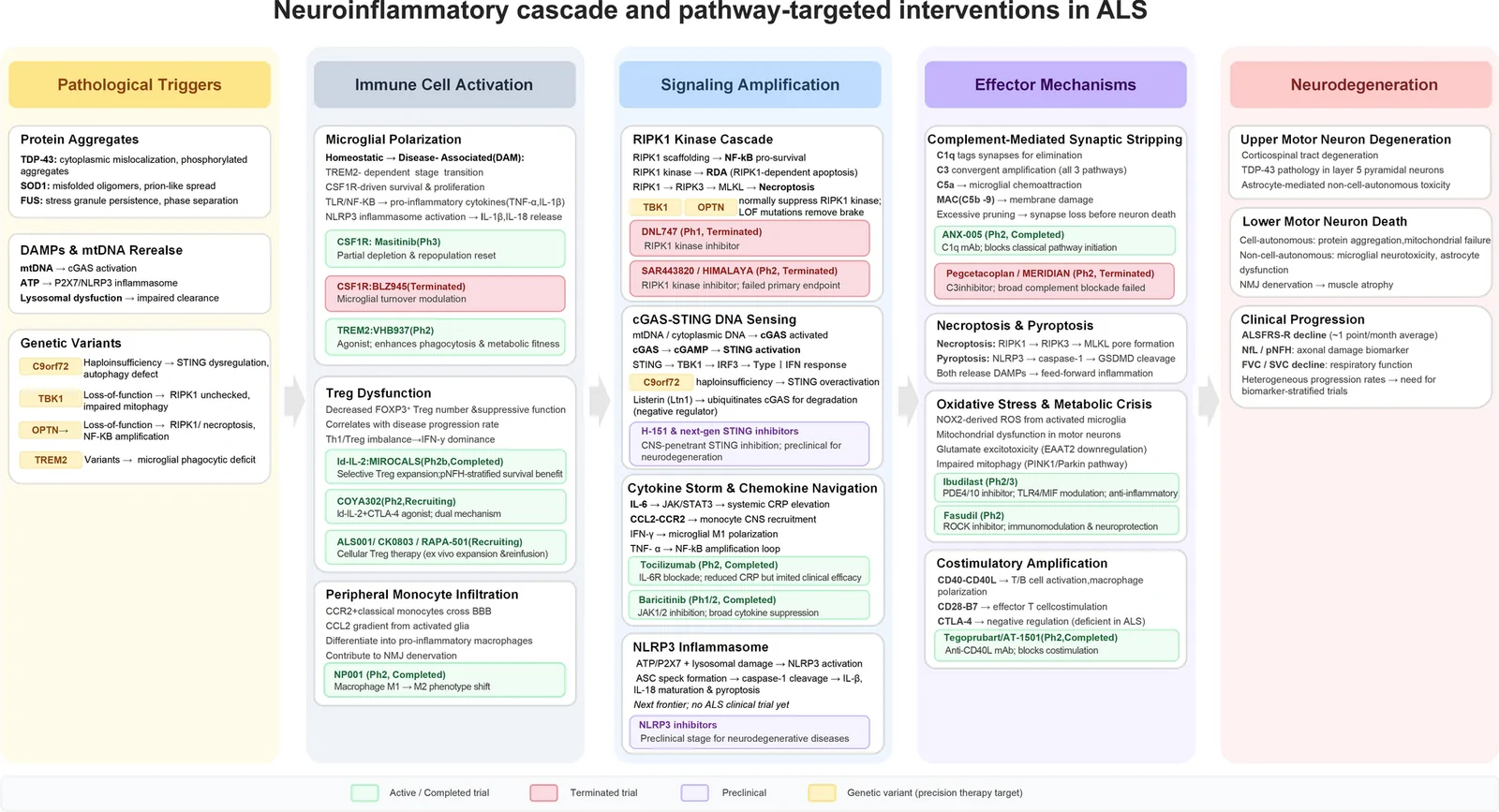
Neuroinflammatory cascade and pathway-targeting interventions in ALS. Protein aggregation (TDP-43, SOD1, FUS), DAMP release (mtDNA, HMGB1, ATP), and mutations in *C9orf72*, *TBK1*, and *OPTN*, initiate a sequential neuroimmune cascade. These signals drive microglial M1 polarization, Treg dysfunction, and CCR2^+^ monocyte infiltration across a disrupted blood–brain barrier. Downstream, five core signaling cascades amplify inflammation: RIPK1-dependent necroptosis, cGAS-STING cytosolic DNA sensing, TLR4/PDE signaling via migration inhibitory factor (MIF), a cytokine storm sustained by IL-1β, TNF-α, IL-6, and IFN-γ, and ROCK-mediated cytoskeletal remodeling. These converge on complement activation (C1q/C3/C5), necroptosis (RIPK3–MLKL), and synaptic stripping, ultimately driving motor neuron death, NMJ denervation, and disease progression. Therapeutic agents targeting each node are color-coded by trial status: green, active or completed; red, terminated; purple, preclinical. ALSFRS-R, ALS Functional Rating Scale-Revised; ASC, apoptosis-associated speck-like protein containing a CARD; B7, B7 costimulatory molecule; C1q, complement component 1q; C3, complement component 3; C5a, complement component 5a; C5b-9, complement component 5b-9; C9orf72, chromosome 9 open reading frame 72; CCL2, C–C motif chemokine ligand 2; CCR2, C–C chemokine receptor type 2; CD40L, CD40 ligand; cGAMP, cyclic GMP-AMP; cGAS, cyclic GMP-AMP synthase; CRP, C-reactive protein; CSF1R, colony-stimulating factor 1 receptor; CTLA-4, cytotoxic T-lymphocyte-associated protein 4; DAMP, damage-associated molecular pattern; EAAT2, excitatory amino acid transporter 2; FOXP3, forkhead box P3; FVC, forced vital capacity; IFN, interferon; IL, interleukin; IRF3, interferon regulatory factor 3; JAK, Janus kinase; LOF, loss-of-function; mAb, monoclonal antibody; MAC, membrane attack complex; MIF, macrophage migration inhibitory factor; MLKL, mixed lineage kinase domain-like; mtDNA, mitochondrial DNA; NfL, neurofilament light chain; NLRP3, NOD-like receptor pyrin domain-containing 3; NMJ, neuromuscular junction; NOX2, NADPH oxidase 2; OPTN, optineurin; P2X7, P2X purinoceptor 7; PDE, phosphodiesterase; Ph1-3, Phase 1-3; PINK1, PTEN-induced kinase 1; pNFH, phosphorylated neurofilament heavy chain; RDA, RIPK1-dependent apoptosis; RIPK1, receptor-interacting protein kinase 1; RIPK3, receptor-interacting protein kinase 3; ROCK, Rho-associated coiled-coil-containing protein kinase; ROS, reactive oxygen species; SOD1, superoxide dismutase 1; STAT3, signal transducer and activator of transcription 3; STING, stimulator of interferon genes; SVC, slow vital capacity; TBK1, TANK-binding kinase 1; TDP-43, transactive response DNA-binding protein 43 kDa; Th1, T helper type 1; TLR, Toll-like receptor; TNF-α, tumor necrosis factor alpha; Treg, regulatory T cell; TREM2, triggering receptor expressed on myeloid cells 2

Biomarkers such as phosphorylated RIPK1 at Ser166 (pS166) provide a pharmacodynamic readout of its activation and inhibitor engagement [139, 153, 159]. Given the mechanistic links between *TBK1* or *OPTN* mutations and RIPK1 dysregulation, patients carrying mutations in these genes are hypothesized to be particularly responsive to RIPK1 inhibition. Recent studies have shown elevated RIPK1 pathway activity in patient-derived cells from these genetic subgroups [156, 160]. However, recent clinical failures (https://trial.medpath.com/news/f322ad98cac0eac9/sanofi-denali-neuro-drug-fails-mid-stage-trial-in-als-ms-study-is) of RIPK1 inhibitors in non-stratified, ALS and multiple sclerosis trials highlight the limitations of a uniform, non-stratified approach. Key challenges include the heterogeneous role of RIPK1 across ALS subtypes and disease stages, the potential for disrupting immune homeostasis, and a likely narrow therapeutic window, particularly in advanced disease. Future efforts are needed for precision and combination strategies, shifting from broad targeting to stage- and subtype-tailored modulation (Fig. 2).

## Emerging strategies for ALS immunotherapy

The clinical landscape for ALS immunotherapy is shifting from broad-spectrum immunosuppression—such as agents like minocycline [161] and corticosteroids [162]—toward precise, mechanism-defined strategies. Key approaches that are under active investigation span four complementary tiers: (1) immune cell-based therapies that directly manipulate adaptive immune populations (Tregs, B cells) or replace immune cell compartments (stem cells); (2) innate immune regulation strategies that modulate the phenotype and function of CNS-resident microglial and infiltrating peripheral macrophages (including CSF1R-mediated microglial turnover, TREM2 agonism, and macrophage activation-state modulation); (3) targeted modulation of specific neuroinflammatory signaling cascades (RIPK1, cGAS–STING, cytokine/complement axes); and (4) delivery (e.g., MR-guided focused ultrasound, NCT02343991) and platform innovations that address the translational bottlenecks common to all of the above. While innate immune sensors such as cGAS–STING and NLRP3 remain in earlier stages of clinical translation, they represent the next frontier, with CNS-penetrant STING inhibitors now in preclinical development for neurodegenerative diseases [163]. The trials (HEALEY ALS Platform Trial, MND-SMART) [164] should be combined with biomarker-enriched precision designs to match the right immunomodulator to the right patient at the right time.

### Targeting adaptive immune

#### Treg augmentation

Tregs are pivotal for maintaining immune tolerance and suppressing excessive inflammation. In ALS patients, both the number and the suppressive function of Tregs are significantly diminished, which correlates with more rapid disease progression [10, 87]. Consequently, enhancing Treg function has emerged as a highly promising therapeutic strategy. Two complementary approaches are being pursued: pharmacological Treg expansion via cytokine-based therapy, and cellular Treg therapy by ex vivo expansion and reinfusion.

Based on the critical role of IL-2 in Treg survival, proliferation, and function, low-dose IL-2 therapy has been tested for Treg enhancement [165]. Administration of ultra-low-dose IL-2 (e.g., Aldesleukin, NCT02059759) can selectively expand the Treg population without broadly activation of effector T cells [166]. The phase 2 MIROCALS trial (NCT03039673) [167] provided pivotal evidence for this approach, demonstrating its safety and a significant increase in peripheral blood Treg proportion. Prespecified adjusted analyses and biomarker stratification showed a survival benefit in a subgroup with low level of CSF phosphorylated-neurofilament heavy-chain, though larger phase 3 trials are needed to confirm the clinical efficacy. A key future direction involves using biomarkers, such as baseline neurofilament or inflammatory signatures, to identify patients most likely to respond. Building on this, COYA 302 therapy (NCT07161999) that combines low-dose IL-2 with a CTLA-4 agonist (a biosimilar for abatacept) to simultaneously expand Tregs and block costimulatory signals driving effector T cell activation, is currently in phase 2 recruitment.

Ex vivo Treg expansion offers a complementary cellular approach. In this approach, Tregs are isolated from a patient via leukapheresis, expanded and functionally enhanced in vitro using factors like IL-2 and rapamycin [168], and then reinfused into the patient. Early-stage clinical trials (ALS001; NCT03241784, NCT04055623) have confirmed the safety and good tolerability of this protocol, with evidence suggesting that the reinfused cells can persist in the body for up to a year [169]. Another autologous hybrid Treg/Th2 cell product, RAPA-501, is undergoing a phase 2/3 expanded access trial (NCT04220190, NCT06169176) in high-risk ALS patients (e.g. SVC < 50%), evaluating the safety and effect on disease progression. Additionally, allogeneic cord blood-derived Tregs engineered with the CRANE technology (CK0803/REGALS; NCT05695521) are designed to home to inflamed CNS tissue via CD11a and CXCR3 expression, and are currently in phase 1/1b evaluation. Key pharmacodynamic readouts across these trials include Treg suppressive function, CSF and plasma inflammatory markers, and neurofilament levels (Table 1).

**Table 1:** Immune cell-targeting and cell-based immunotherapy trials in ALS

Cell type & source	Product name	Target or mechanism	Sponsor	Phase	NCT#	Key pharmacodynamics/biomarkers
*Pharmacological Treg support (IL-2)*						
Pharmacological Treg expansion	Low-dose IL-2 (IMODALS)	IL-2 (selective Treg expansion)	Multicenter academic	Phase 2a	NCT02059759	Treg %; NfL; safety
Pharmacological Treg expansion	Low-dose IL-2 (MIROCALS)	IL-2 (selective Treg expansion)	Multicenter academic	Phase 2b	NCT03039673	Treg %; pNFH; survival (pre-specified subgroup)
Pharmacological Treg expansion + co-stimulation blockade	COYA 302	Low-dose IL-2 + abatacept biosimilar (DRL_AB)	Coya Therapeutics	Phase 2	NCT07161999	Treg %; cytokines (IL-6, TNF-α); ALSFRS-R
*Treg cell therapies*						
Autologous expanded Tregs	ALS001	Restores suppressive function; low-dose IL-2 co-support	Houston Methodist / Coya Therapeutics	Phase 1; Phase 2a	NCT03241784; NCT04055623	Treg suppressive function; oxidative stress markers; IL-2 PK
Allogeneic cord blood Tregs	CK0803 (REGALS)	CRANE-engineered homing via CD11a/CXCR3 to inflamed CNS	Cellenkos Inc	Phase 1/1b	NCT05695521	Safety (DLT); persistence/homing; CSF and plasma inflammatory markers; NfL
Autologous hybrid Treg/Th2 T stem cells	RAPA-501	Epigenetic reprogramming; dual suppressive & anti-inflammatory cytokine secretion	RAPA Therapeutics	Phase 2/3	NCT04220190; Expanded access NCT06169176	Treg/Th2 and Th1 frequency; cytokines (IL-6, TNF-α); ALSFRS-R
*Innate immune / microglial modulation*						
CSF1R inhibition (microglial depletion/repopulation)	BLZ945 (Sotuletinib)	CSF1R (microglial survival/renewal dynamics)	Novartis	Phase 2	NCT04066244	Microglial activation markers; ALSFRS-R
Multi-target TKI (includes CSF1R activity)	Masitinib	CSF1R + additional kinase targets	AB Science	Phase 2; Phase 3; Phase 3	NCT02588677; NCT03127267; NCT07174492	ALSFRS-R; survival; inflammatory markers
TREM2 agonist antibody	VHB937 (ASTRALS)	TREM2 (microglial phagocytosis & metabolic fitness)	Novartis	Phase 2	NCT06643481	Microglial activation; CSF biomarkers; ALSFRS-R
Macrophage activation modulation	NP001 (Sodium Chlorite)	Macrophage activation state	Neuraltus Pharmaceuticals	Phase 2	NCT02794857	Systemic inflammatory markers; ALSFRS-R
*Stem cell therapies*						
Autologous MSC-NTF	NurOwn	Immunomodulation via neurotrophic & anti-inflammatory factors	BrainStorm Cell Therapeutics	Phase 2; Phase 3; Phase 3b	NCT02017912; NCT03280056; NCT06973629; Expanded access NCT04681118	CSF biomarker shifts (MCP-1, CHIT1/YKL-40, VEGF); ALSFRS-R response
Autologous BM-MSC	Neuronata-R (Lenzumestrocel)	Neurotrophic support and anti-inflammatory paracrine signaling	CorestemChemon Inc	Phase 3	NCT04745299	NfL; MCP-1; ALSFRS-R & CAFS scores

#### B cell and Breg modulation

The traditional view of B cells in ALS primarily focused on their pathogenic role through autoantibody production. However, recent research has unveiled anti-inflammatory and neuroprotective functions of specific B cell subsets, particularly Bregs, leading to a paradigm shift in therapeutic thinking. Preclinical studies have shown that infusing healthy donor-derived B cells into SOD1^G93A^ ALS mice significantly delays disease onset, improves motor function, and extends survival. These effects are attributed primarily to the Breg-mediated immunomodulation through IL-10 secretion rather than antibody production [93]. A case report described short-term clinical improvement and reduced inflammatory markers in a patient with advanced ALS following allogeneic B cell infusion [93], providing preliminary proof-of-concept, albeit with limited evidence. Dedicated clinical trials for B cell/Breg therapy in ALS are not yet registered, and this approach remains at the translational frontier (Table 1).

#### Stem cell-based immune reset

Driven by the hypothesis that resetting the immune landscape could slow neurodegeneration, hematopoietic stem cell transplantation (HSCT) has been investigated in ALS. An early allogeneic HSCT study in six patients revealed donor-cell engraftment in the CNS but failed to yield clinical benefit [170]. A subsequent phase 1/2a trial of autologous HSCT enrolled 11 patients (8 treated); while the safety profiles were acceptable, the intervention did not significantly alter disease trajectories [171]. In parallel, mesenchymal stem cell (MSC) modalities have demonstrated safety and elicited biomarker responses. Repeated intrathecal administration of MSCs [172] and neurotrophic factor-secreting MSCs (NurOwn, BrainStorm Cell Therapeutics) [173] have progressed through phase 2 and phase 3 trials (NCT02017912; NCT03280056; NCT06973629), with observation of CSF biomarker shifts (MCP-1, CHIT1/YKL-40, VEGF [vascular endothelial growth factor]), yet robust clinical efficacy remains elusive. Neuronata-R (lenzumestrocel, CorestemChemon), an autologous bone marrow-derived MSC product, is currently in phase 3 evaluation (NCT04745299), with NfL as an exploratory endpoint (Table 1).

### Targeting innate immunity

#### Microglial phenotype modulation

Microglia are central executors of neuroinflammation. Current therapeutic strategies aim not to eliminate them entirely [174]*—*which could be detrimental*—*but to “reprogram” their functional phenotype, shifting them from a pro-inflammatory, neurotoxic state (historically labeled M1-like) towards an anti-inflammatory, protective/reparative state (M2-like). It is important to note that in vivo microglial states are very complex [115, 175], far beyond a simple M1/M2 dichotomy [39, 176]. Two principal strategies are being pursued: CSF1R-mediated microglial turnover and TREM2-driven metabolic and phagocytic reactivation.

CSF1R is essential for microglial survival and renewal [177, 178]. Short-term use of CSF1R inhibitors can partially deplete brain microglia; the subsequent repopulating cell population may contribute to re-establishment of a more homeostatic cellular environment [179, 180]. However, long-term or extensive inhibition may impair the normal physiological functions of microglia, requiring careful consideration. CSF1R-directed agents in ALS clinical trials include the selective inhibitor BLZ945 (Sotuletinib; NCT04066244; terminated) and the multi-target tyrosine kinase inhibitor masitinib (NCT02588677, NCT03127267, NCT07174492), which has shown more promising results in phase 2/3 studies. These agents seek to reset microglial survival and renewal dynamics, potentially shifting disease-associated microglia towards homeostasis.

TREM2 is a key receptor regulating microglial metabolism, phagocytosis, and inflammatory responses [181]. TREM2-targeting drug development is more advanced in Alzheimer’s disease, with the small-molecule agonist VG-3927 [182] having entered clinical trials (NCT06343636). In ALS, the TREM2 agonist antibody VHB937 (ASTRALS, NCT06643481) has progressed to a Phase 2 clinical trial for ALS. Activating the TREM2 pathway holds particular promise for ALS patients harboring specific genetic variants, such as those in C9orf72 [41, 183] or TREM2 [184] itself, as it may enhance the microglial clearance of pathological protein aggregates and suppress detrimental neuroinflammatory responses (Table 1).

#### Macrophage modulation

Beyond microglia, peripheral macrophages represent another innate immune target. NP001 (Sodium Chlorite; NCT02794857) modulates macrophage activation states and has been evaluated in a phase 2 trial. Additionally, tegoprubart (AT-1501-A201; NCT04322149), an anti-CD40 ligand monoclonal antibody that targets CD4^+^ T cells and B cells while also modulating macrophage and microglial activation, has completed a phase 2 trial in ALS. These approaches reflect the growing recognition that innate immune cells at both the central and the peripheral levels contribute to disease propagation and may be amenable to targeted pharmacological intervention (Table 1).

### Targeting inflammatory signaling cascades

Beyond the cell-level interventions, several discrete intracellular and extracellular signaling pathways have been identified as tractable therapeutic targets in ALS (Table 2).

**Table 2:** Signaling pathway-targeting trials in ALS

Group	Drug name	Target or pathway	Sponsor	Phase	NCT#	Trial status
RIPK1 inhibition (Inflammation/Necroptosis)	DNL747	RIPK1 (Inflammation/Necroptosis)	Sanofi	Phase 1	NCT03757351	Terminated
	SAR443820(DNL788)	RIPK1 (Inflammation/Necroptosis)	Sanofi	Phase 2	NCT05237284	Terminated
PDE/MIF/TLR4 modulation	Ibudilast (MN-166)	Phosphodiesterase inhibitor	MediciNova	Phase 1/2, 2/3	NCT02714036; NCT04057898	Completed; Not yet recruiting
ROCK inhibition	Fasudil (WP-0512)	Rho kinase (ROCK) inhibitor	Woolsey Pharmaceuticals	Phase 2	NCT05218668	Not yet recruiting
Complement C1q inhibition	ANX-005	C1q-specific mAb	Annexon	Phase 2	NCT04569435	Completed
Complement C3 inhibition	Pegcetacoplan	Complement C3 inhibitor	Apellis Pharmaceuticals	Phase 2	NCT04579666	Terminated
IL-6R blockade	Tocilizumab	IL-6R	Multicenter academic collaboration	Phase 2	NCT02469896	Completed
CD40L blockade	Tegoprubart (AT-1501-A201)	Anti-CD40 ligand mAb that targets CD4T cells and B cells	Eledon Pharmaceuticals	Phase 2	NCT04322149	Completed
JAK pathway inhibition	Baricitinib (NCB28050)	JAK signaling	Multicenter academic collaboration	Phase 1/2	NCT05189106	Completed

#### RIPK1/necroptosis pathway

Targeting the RIPK1 kinase, a link between inflammation and necroptosis [139], has faced clinical challenges despite strong preclinical rationale. Its upregulation has been observed in ALS spinal cords driving microglial activation and neurodegeneration [140]. Despite strong preclinical rationale, clinical translation has been challenging: the phase 1 program with DNL747 (NCT03757351) and the phase 2 HIMALAYA trial of SAR443820/DNL788 (NCT05237284) were both terminated, with the latter failing to meet its primary endpoint. These outcomes underscore the necessity for precise patient stratification—potentially focusing on genetic backgrounds such as *TBK1* or *OPTN* mutations—and robust biomarker confirmation of target engagement in future trials.

#### cGAS–STING pathway

The cGAS–STING pathway is an emerging target with high potential. Its activation occurs in neurons in response to DNA damage from ALS pathology, and its inhibition rescues neurodegeneration in models [123]. Pharmacological inhibition of STING (e.g., with H-151) has been shown to suppress these innate immune markers and rescue neurodegeneration in ALS models [185]. Furthermore, the E3 ubiquitin ligase Listerin has been identified as a critical negative regulator of this pathway. Listerin [186] promotes the ubiquitination and ESCRT-mediated lysosomal degradation of cGAS, and its deficiency exacerbates neuroinflammation and motor deficits. While not yet in ALS clinical trials, its translation will require innovative delivery strategies for CNS engagement and biomarker-enriched trial designs.

#### Cytokine signaling axes

The IL-6 axis presents a complex target. IL-6R blockade (tocilizumab) [187] effectively reduces systemic C-reactive protein (CRP) levels in ALS patients but has shown limited clinical efficacy. In addition, IL-6 deficiency in mouse models [188] failed to alter disease progression, suggesting that IL-6 may be a marker rather than a primary driver of neurodegeneration in ALS. CD40L blockade with tegoprubart (AT-1501-A201, NCT04322149) targets the CD4 T cell–B cell co-stimulatory axis and has completed phase 2 evaluation. JAK pathway inhibition with baricitinib (NCT05189106) addresses the convergent downstream signaling of multiple cytokines and has completed a phase 1/2 trial.

#### Chemokine axis

The CCL2–CCR2 axis drives peripheral CCR2^+^ monocyte recruitment into the CNS parenchyma and peripheral nerves, contributing to NMJ denervation. Local neutralization of CCL2 has been found to preserve NMJ integrity and reduce immune infiltration [145, 189], providing rationale for clinical targeting of this axis.

#### Complement system

Complement dysregulation couples synaptic tagging and inflammatory amplification. Clinical C1q inhibition with ANX-005 (NCT04569435, completed) and C3 inhibition with pegcetacoplan (NCT04579666, terminated due to lack of efficacy) can interrupt this cascade. The termination of the MERIDIAN trial of pegcetacoplan highlights the challenge of complement inhibition in a heterogeneous patient population without prospective biomarker stratification.

#### PDE/TLR4 and Rho kinase (ROCK) inhibition

Ibudilast (MN-166), a phosphodiesterase inhibitor that also downregulates TLR4 signaling and macrophage MIF (migration inhibitory factor), has completed a phase 1/2 trial (NCT02714036), and a phase 2/3 program (NCT04057898) is not yet recruiting. Fasudil (WP-0512), a ROCK inhibitor with anti-neuroinflammatory and neuroprotective properties, is under phase 2 evaluation (NCT05218668).

### Emerging delivery and platform strategies

Realizing the therapeutic potential of the strategies described above requires adequate CNS drug exposure and identification of the right patients. MR-guided focused ultrasound (MRgFUS, NCT02343991) offers a non-invasive approach to transiently open the BBB, potentially enhancing CNS delivery of small molecules that otherwise fail to achieve therapeutic concentrations in the spinal cord and motor cortex. At the trial design level, adaptive platform trials—exemplified by the HEALEY ALS Platform Trial and MND-SMART—employ a shared, concurrent control arm across multiple investigational agents, thereby substantially reducing the total sample size required per intervention. Adoptive randomization redirects enrollment toward agents showing preliminary signal and away from those failling [164]. Finally, a proof-of-concept from a genetically defined microgliopathy offers a mechanistic reference point, though not a directly translatable model, for CNS immune cell replacement. In adult-onset leukoencephalopathy with axonal spheroids and pigmented glia (ALSP, a monogenic CSF1R disorder in which microglial dysfunction is the primary, cell-autonomous disease driver), bone marrow transplatation arrested disease progression in eight patients [190]. The relevance to ALS is constrained by a fundamental pathogenic difference: ALSP is a single-gene, cell-autonomous microgliopathy in which correcting the defective microglial compartment addresses the root cause, whereas ALS is multifactorial and non-cell-autonomous, with neuroinflammation representing one convergent mechanism. The ALSP result therefore establishes the biological principle that CNS-resident immune cells can be replaced in humans with measurable clinical effect, and provides a rationale—rather than direct evidence—for evaluating microglia-directed strategies in biomarker-stratified ALS subgroups with predominant neuroinflammatory burden.

## Inflammatory biomarkers as the cornerstone of precision medicine

### Biomarkers for patient stratification and treatment monitoring

The use of inflammatory biomarkers is transforming ALS immunotherapy trials from empirical screening to proof-of-biology. These markers can not only stratify patients into immunologically defined subgroups before treatment, but also verify target engagement and biological response after treatment initiation.

CSF provides the most direct window into immune-degenerative processes within the CNS [141, 191]. The chitinase family proteins CHIT1 and CHI3L1/YKL-40 are the most extensively validated glial biomarkers in ALS. CHIT1 is predominantly secreted by activated microglia and macrophages, whereas CHI3L1 is primarily astrocyte-derived [192, 193]. Elevated CSF CHIT1 is strongly associated with faster disease progression and shorter survival, making it a candidate indicator for identifying subgroups of high inflammation [194]. CSF CCL2/MCP-1 further correlates with microglial activation in the motor cortex and predicts worse clinical trajectories [192]. Composite signatures combining CHIT1, CCL2, and other inflammatory mediators have been proposed to enhance stratification precision [195]. Critically, these markers serve a pharmacodynamic function after treatment: reductions in CSF CHIT1 and CHI3L1 have been demonstrated following treatment with the anti-CD40L antibody tegoprubart (NCT04322149), providing direct evidence of target engagement within the CNS immune compartment [196]. These dual roles position CSF chitinases as anchor biomarkers for mechanism-based trial design.

Peripheral blood biomarkers capture the systemic dimension of immune dysregulation in ALS and offer practical advantages in longitudinal monitoring. Elevated CRP and neutrophil-to-lymphocyte ratio reflect systemic inflammatory burden and correlate with accelerated disease progression and poor survival [104, 197]. Neurofilament light chain (NfL), measurable in both plasma and CSF, has emerged as a cross-trial pharmacodynamic standard: its stabilization or reduction in the trials of CNM-Au8 [198, 199] and tofersen [200] indicates attenuation of axonal injury, and blood NfL has been incorporated as a pharmacodynamic endpoint in the HEALEY ALS Platform Trial. The number and the suppressive function of Tregs constitute a distinct peripheral immune readout: reduced Treg frequency and impaired suppressive capacity are associated with faster progression [75, 87]. These metrics are increasingly used as pharmacodynamic biomarkers in adaptive immune trials. In the MIROCALS low-dose IL-2 trial, for example, immune profiling confirmed that IL-2 selectively expanded the Treg compartment, providing direct evidence of target engagement [167]. Together, these peripheral markers enable non-invasive, longitudinally feasible monitoring that complements the mechanistic depth of CSF profiling.

Molecular neuroimaging with translocator protein (TSPO)-positron emission tomography (PET) tracers, including the first-generation [^11^C]PBR28 and the higher-affinity second-generation [^18^F]GE-180, enables in vivo visualization of glial activation in the motor cortex and spinal cord [201, 202]. Beyond its prognostic value, TSPO-PET has been incorporated as a target engagement readout in interventional trials: BLZ945 (NCT04066244) and MN-166/ibudilast (NCT02714036) both used TSPO-PET to confirm CNS targeting by the intervention. As a pharmacodynamic marker, phosphorylated RIPK1 (pS166) is elevated in ALS CNS tissue [139] and is under active investigation as a CSF readout for RIPK1 pathway engagement—an example of how emerging pathway-specific markers may extend the target engagement toolkit beyond glial activation. The spatial resolution of TSPO-PET is particularly relevant for patient stratification: the regional differences in neuroinflammatory burden between motor cortex and spinal cord may reflect distinct disease stages or mechanistic subtypes, a dimension that warrants prospective investigation.

When used in combination, these biomarkers can further reveal a consistent pattern of inter-patient variability in inflammatory burden, glial activation state, and adaptive immune status that cannot be explained by clinical phenotype alone. Patients with high CSF CHIT1 and TSPO-PET positivity do not necessarily share the same peripheral immune profile as those with Treg exhaustion and systemic inflammation; patients with distinct genetic backgrounds exhibit immune signatures that diverge qualitatively, not merely quantitatively, from the sporadic ALS population [122]. This biological heterogeneity is not merely a statistical nuisance to be controlled for trial design, but a central obstacle to immunotherapy efficacy. This raises a question that the available biomarker toolkits have not yet been designed to answer: whether distinct, reproducible immune subgroups exist in ALS patients that are stable enough to guide treatment selection, and whether matching mechanism-specific interventions to such subgroups can improve outcomes. Addressing this question requires a conceptual framework that goes beyond individual biomarker performance. Consequently, the framework of neuroimmune endotypes are raised and examined in the following section.

### Toward neuroimmune endotypes: an unresolved but necessary framework

Converging evidence across multiple levels of analysis supports that the heterogeneity described above reflects mechanistically distinct disease states, rather than being a random biological noise. Post-mortem transcriptomic studies have identified at least three reproducible molecular subtypes of ALS, ALS-Glia subtype, oxidative stress (ALS-Ox) subtype and TDP-43 pathology (ALS-TE) subtype. The ALS-Glia subtype is defined by microglial activation and neuroinflammatory signatures that correlates with disease duration in the spinal cord [203, 204]. At the genetic level, *C9orf72* loss-of-function leads to STING hyperactivation and an elevated type I interferon signature detectable in peripheral blood, a distinct immunophenotype absent in sporadic ALS [122]. T cell immunophenotyping at diagnosis has further identified patient subgroups with divergent immune profiles that predict differential rates of disease progression and survival [75]. Collectively, these converging lines of evidence establish that ALS neuroinflammation is not a monolithic process but involves a set of mechanistically distinct states. Current trial designs, which enroll immunologically undifferentiated populations, are structurally unable to resolve this biological reality.

Neuroimmune “endotypes” are disease subtypes defined by shared molecular mechanisms rather than clinical phenotype. This concept offers a potential organizing framework for this heterogeneity. In respiratory medicine, endotype-guided therapy has transformed the management of severe asthma. Two principal endotypes have been defined: eosinophilic and neutrophilic, each with a distinct biomarker signature. This distinction now directs the selection of biologic agents, leading to substantially improved outcomes [205, 206]. Translating this paradigm to ALS is mechanistically compelling but currently premature [13, 207]. Unlike asthma, where a single fluid biomarker (blood eosinophil count) can reliably stratify patients, ALS neuroimmune heterogeneity likely requires multimodal characterization, including CSF inflammatory profiling, peripheral immune cell phenotyping, molecular imaging, and genetic testing, none of which has been prospectively validated as a stratification tool in an interventional trial. Critically, the molecular subtypes identified in post-mortem tissue cannot yet be mapped onto living patients through accessible biomarkers, leaving a fundamental gap between the biological evidence for heterogeneity and the clinical infrastructure needed to act on it.

Three obstacles currently stand between the endotype concept and clinical practice. The first is a fundamental measurement problem. The molecular subtypes identified in post-mortem tissue have not been shown to correspond to stable, accessible signatures in living patients, and it remains unclear whether any such signatures would be precise enough to guide individual treatment decisions rather than simply describe population-level trends. The second obstacle is the definition. Asthma endotypes can be anchored to a single parameter, blood eosinophil count, which is cheap, reproducible, and actionable. In contrast, ALS immune stratification requires integration of CSF inflammatory profiles, peripheral immune phenotyping, molecular imaging, and genetic context, a combinatorial demand that no prospective study has yet attempted to operationalize. The third gap is the most consequential: no completed trials of ALS immunotherapy have pre-specified immune subgroups before enrollment, matched interventions to those subgroups on mechanistic grounds, and used pharmacodynamic readouts to confirm that the drug reached and engaged its target before assessment of patient outcomes. Currently, neuroimmune endotypes are best understood as a working hypothesis, and should not be considered as a classification system ready for use to guide clinical decisions.

## Challenges and prospectives

While immunotherapy represents a promising frontier for ALS, clinical translation has been impeded by specific gaps that need to be addressed in future trials.

The first is the narrow therapeutic window. Cortical hyperexcitability and neuroimmune activation are early drivers of pathology, often evident in the presymptomatic phase long before clinical diagnosis [208–210]. By the time of patient enrollment, substantial motor neuron loss has already occurred. The inconsistency between preclinical studies routinely beginning treatment at or before symptom onset and clinical trials enrolling patients after neuronal damage, is a fundamental reason why interventions that work in animal models have repeatedly failed in humans [211, 212].

The second is inadequate verification of target engagement. Most early immunotherapy trials enrolled patients and administered treatment without confirming that the drug reached and engaged its intended CNS target. This makes it impossible to distinguish true biological failure from pharmacokinetic failure [213]. The CNS immune landscape further compounds this problem: blocking one pathway often triggers compensatory upregulation of others [214, 215], an effect that is difficult to anticipate without longitudinal immune profiling. The minocycline Phase III trial (*n* = 412) illustrates that the drug not only failed to slow progression, but accelerated functional decline (ALSFRS-R slope: − 1.30 vs. − 1.04/month, *P* = 0.005) [161]. This outcome was consistent with a compensatory pro-inflammatory rebound in a population whose immune state was not characterized at baseline.

The third is the unaddressed patient heterogeneity. ALS is not immunologically uniform: *C9orf72* loss-of-function drives STING hyperactivation and a type I interferon signature, which are absent in most sporadic cases [122], while *SOD1* mutations are associated with distinct microglial activation patterns [216]. Trials that enroll undifferentiated populations without mechanistic stratification may dilute treatment signals of a genuinely effective intervention.

**Fig. 3:**
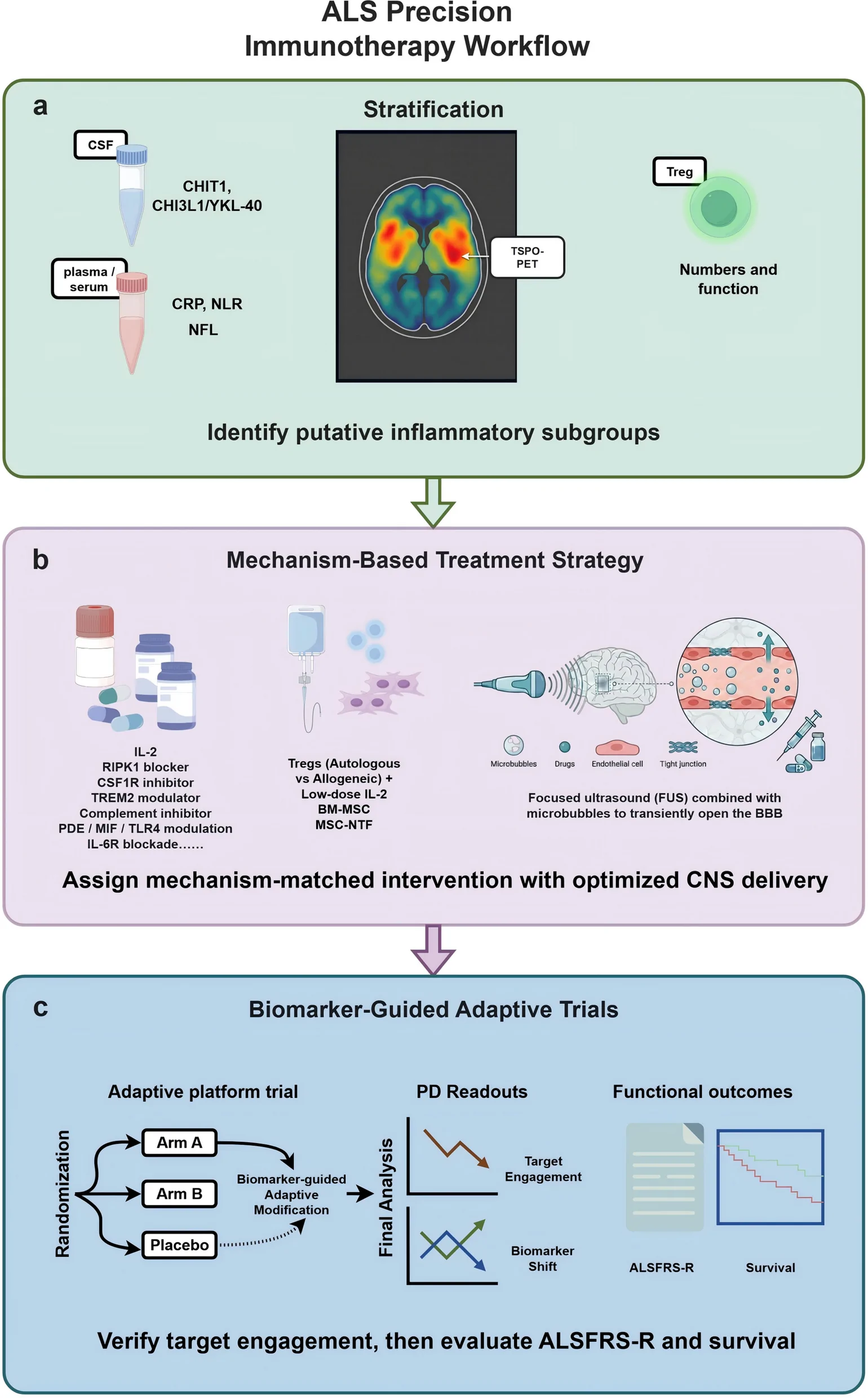
Precision immunotherapy workflow for ALS. A proposed three-stage framework for precision immunotherapy in ALS. **a** Stratification. Patients are profiled using a multimodal biomarker panel to identify putative immune subgroups. CSF markers (CHIT1, CHI3L1/YKL-40) reflect CNS glial activation; plasma markers (CRP, neutrophil-to-lymphocyte ratio [NLR], NfL) capture systemic inflammation and axonal injury; TSPO-PET provides spatial quantification of glial activation in the motor cortex and spinal cord; and Treg cell numbers and suppressive function complete the peripheral immune profile. **b** Mechanism-based treatment strategy. Candidate interventions are selected according to the dominant immune mechanism identified in Stage **a**. Investigational agents include small molecules and biologics targeting innate immunity (RIPK1 blockers, CSF1R inhibitors, TREM2 modulators, complement inhibitors, IL-6R blockade) and adaptive immunity (low-dose IL-2). Cell-based approaches include Treg infusion and MSC-derived therapies. Focused ultrasound (FUS) with microbubbles provides spatially targeted BBB opening to enhance CNS drug delivery. **c** Biomarker-guided adaptive trial. Pharmacodynamic readouts regarding target engagement and biomarker shifts, are verified before interpreting functional outcomes (ALSFRS-R, survival), enabling distinction of biological failure from pharmacokinetic or patient-selection failure

We propose three strategies to address the failure modes correspondingly. First, standardized biomarker panels for patient stratification must be prospectively validated. CSF chitinases (CHIT1, CHI3L1/YKL-40) [80, 194] and TSPO-PET [7] show promise in identifying high-inflammation-level subgroups and should be incorporated as pre-specified stratification criteria in future trials. Emerging pathway-specific markers including HERV-K env expression [124, 217] and RIPK1 phosphorylation [139, 159] require further clinical validation before they can guide treatment allocation. Second, CNS drug delivery must be optimized to ensure target engagement. BBB and BSCB alterations in ALS unpredictably alter CNS drug exposure [218]. Delivery technologies such as MR-guided focused ultrasound with microbubbles [219, 220], offer a spatially targeted means to transiently enhance penetration in the motor cortex and spinal cord. Third, for endotype–treatment matching, trials must pre-specify immune subgroups as enrollment criteria, assign mechanism-matching interventions, and confirm target engagement pharmacodynamically before interpreting clinical outcomes (Fig. 3). Third, trial infrastructure capable of managing biological complexity at scale is needed. The HEALEY ALS Platform Trial exemplifies the power of adaptive, biomarker-enriched methodologies. By screening multiple agents simultaneously, pre-specifying pharmacodynamic endpoints (e.g., blood NfL) [221], and enabling rapid go/no-go decisions, platform trials can efficiently identify which mechanism-defined interventions would benefit which patient subgroups.

## Conclusion

In summary, neuroinflammation in ALS is a mechanistically heterogeneous, temporally dynamic process that actively drives disease progression. Prior immunotherapies have failed largely because they did not engage this heterogeneity on its own terms. Convergent evidence supports the existence of distinct immune states, but the neuroimmune endotype concept remains a working hypothesis rather than a validated classification: molecular subtypes identified in post-mortem tissue have not yet been mapped onto accessible biomarkers in living patients, and no trial has prospectively tested mechanism-matched interventions in defined subgroups. With validated fluid biomarkers and adaptive platform trial infrastructure now available, the question is no longer whether neuroinflammation should be targeted in ALS, but whether the field is prepared to design trials that can answer it.

## Data Availability

No datasets were generated or analysed during the current study.

## Abbreviations

ALS: Amyotrophic lateral sclerosis
BBB: Blood–brain barrier
BSCB: Blood-spinal cord barrier
CCL: C-C motif chemokine ligand
CHIT1: Chitotriosidase
CNS: Central nervous system
CSF: Cerebrospinal fluid
CSF1R: Colony stimulating factor 1 receptor
CX3CL1: C-X3-C motif chemokine ligand 1
DAM: Disease-associated microglia
DAO: Disease-associated oligodendrocyte
HSCT: Hematopoietic stem cell transplantation
IFN: Interferon
IL: Interleukin
MHC: Major histocompatibility complex
MSC: Mesenchymal stem cell
NK: Natural killer cells
NLRP3: NOD-like receptor family pyrin domain containing 3
NMJ: Neuromuscular junction
OPC: Oligodendrocyte precursor cell
PET: Positron emission tomography
PRR: Pattern recognition receptor
RIPK1: Receptor-interacting serine/threonine-protein kinase 1
ROS: Reactive oxygen species
SOD1: Superoxide dismutase 1
STING: Stimulator of interferon genes
TBK1: TANK-binding kinase 1
TDP-43: TAR DNA-binding protein 43
TLR4: Toll-like receptor 4
TSPO: Translocator protein
